# Interface Engineering of Fe_7_S_8_/FeS_2_ Heterostructure in situ Encapsulated into Nitrogen-Doped Carbon Nanotubes for High Power Sodium-Ion Batteries

**DOI:** 10.1007/s40820-023-01082-w

**Published:** 2023-04-30

**Authors:** Penghao Song, Jian Yang, Chengyin Wang, Tianyi Wang, Hong Gao, Guoxiu Wang, Jiabao Li

**Affiliations:** 1 College of Chemistry and Chemical Engineering, Yangzhou University, 180 Si-Wang-Ting Road, Yangzhou, 225002 Jiangsu People’s Republic of China; 2 Centre for Clean Energy Technology, Faculty of Science, University of Technology Sydney, PO Box 123, Broadway, NSW, 2007 Australia; 3 Key Lab of Fluorine and Silicon for Energy Materials and Chemistry of Ministry of Education, College of Chemistry and Chemical Engineering, Jiangxi Normal University, Nanchang, 330022 People’s Republic of China

**Keywords:** Iron sulfides, Heterostructure, Nitrogen-doped carbon nanotubes, Ester-based electrolyte, Ether-based electrolyte

## Abstract

Heterostructure engineering combined with carbonaceous materials shows great promise toward promoting sluggish kinetics, improving electronic conductivity, and mitigating the huge expansion of transition metal sulfide electrodes for high-performance sodium storage. Herein, the iron sulfide-based heterostructures in situ hybridized with nitrogen-doped carbon nanotubes (Fe_7_S_8_/FeS_2_/NCNT) have been prepared through a successive pyrolysis and sulfidation approach. The Fe_7_S_8_/FeS_2_/NCNT heterostructure delivered a high reversible capacity of 403.2 mAh g^−1^ up to 100 cycles at 1.0 A g^−1^ and superior rate capability (273.4 mAh g^−1^ at 20.0 A g^−1^) in ester-based electrolyte. Meanwhile, the electrodes also demonstrated long-term cycling stability (466.7 mAh g^−1^ after 1,000 cycles at 5.0 A g^−1^) and outstanding rate capability (536.5 mAh g^−1^ at 20.0 A g^−1^) in ether-based electrolyte. This outstanding performance could be mainly attributed to the fast sodium-ion diffusion kinetics, high capacitive contribution, and convenient interfacial dynamics in ether-based electrolyte.

**Supplementary Information:**

The online version contains supplementary material available at 10.1007/s40820-023-01082-w.

## Highlights

- Iron sulfide-based heterostructure in situ hybridized with nitrogen-doped carbon nanotubes was prepared through a successive pyrolysis and sulfidation approach.
- The as-prepared Fe_7_S_8_/FeS_2_/NCNT electrode exhibits superior sodium storage performance in both ester and ether-based electrolytes.
- The structure advantages of the electrode contribute to high electrochemical performance in the ester-based electrolyte, while fast ionic diffusion and favorable capacitive behavior result in the robust sodium storage performance in the ether-based electrolyte.

**Supplementary Information:**

The online version contains supplementary material available at 10.1007/s40820-023-01082-w.

## Graphical abstract

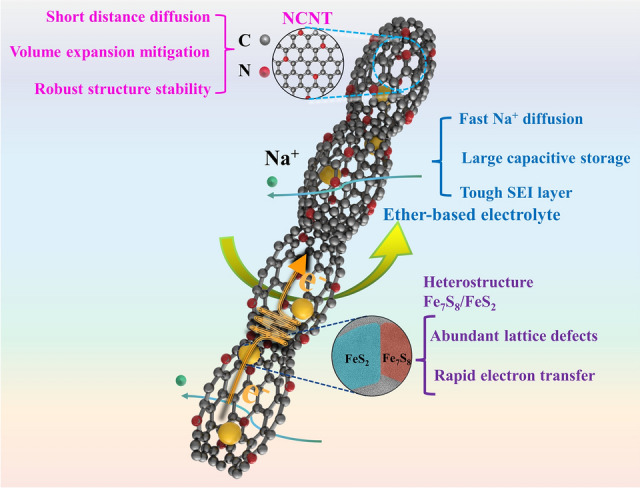

## Introduction

Sodium-ion batteries (SIBs) with a similar operating mechanism to lithium-ion batteries and abundant sodium resources are considered as one of the most promising candidates for low-cost renewable energy storage [1–5]. However, sodium ion has a relatively large ionic radius and sluggish reaction kinetics, which inevitably lead to capacity degradation and cycling instability, hindering the practical application of SIBs [6–10]. Therefore, it is of great significance to develop novel electrode materials with high capacity and long cycle life.

Transition metal sulfides (TMSs) are considered as promising electrode candidates due to their high theoretical capacities [11–15]. The weak M-S bond is favorable for electrochemical conversion reaction, resulting in active redox kinetics and reversibility. As a typical TMSs, pyrite FeS_2_ with a high theoretical capacity (894 mAh g^−1^), low cost, and environmental friendliness, is a promising and effective sodium storage electrode material [16–19]. However, similar to other metal sulfides, the iron sulfides-based electrodes suffer from poor electronic conductivity and huge volume change upon cycling, resulting in sluggish Na^+^ insertion/extraction, large electrochemical polarization, electrode degradation, and hence poor rate capability and limited cycling life [20–23].

To overcome the above mentioned challenges, many efforts have been made, mainly focusing on several aspects [24–27]. These include: (i) Combining with conductive matrix to accelerate electron transport and alleviate the structure expansion of the active material during cycling; (ii) Creating a large number of lattice defects to provide abundant active sites; (iii) Optimizing the electrolyte to facilitate Na^+^ diffusion kinetics, improve the capacitive contribution of charge storage, and form robust solid electrolyte interface- (SEI) layer. Liu et al. [28] designed FeS_2_ nanocrystals wrapped in a turbostratic carbon framework (FeS_2_–C) via a mechanical alloying process. The FeS_2_–C electrode achieved rapid electrons/ions transport and robust structural stability, thus enabling improved rate capability and long cycling performance for SIBs. Chen et al. [29] designed Fe_1−x_S/MoS_2_ heterostructure with abundant active sites, and the corresponding electrode exhibited good rate capability and stable cycling performance. In addition to improving the sodium storage performance from the perspective of material synthesis, it was also found that different electrolytes can greatly affect the structural transformation and electrochemical performance of FeS_2_-based anodes during cycling. Zhou et al. [30] demonstrated that the FeS_2_@C electrode coupled with ether-based electrolyte could realize high ionic diffusion and electrochemical reversibility. These individual modification strategies can indeed modify the performance of iron chalcogenide-based electrodes. Therefore, the combination of the above three strategies will simultaneously endow the electrodes with high conductivity, rapid charge transfer, and favorable interfacial behaviors.

Herein, we rationally designed and prepared iron sulfide-based heterostructures encapsulated in N-doped carbon nanotubes (Fe_7_S_8_/FeS_2_/NCNT) via an in situ pyrolysis and sulfidation strategy. The as-prepared Fe_7_S_8_/FeS_2_/NCNT heterostructures inherit substantial lattice defects and sufficient buffer space offered by the NCNT, which endow the Fe_7_S_8_/FeS_2_/NCNT electrode with high sodium storage activity, rapid ionic diffusion kinetics, and excellent electrochemical reversibility. Interestingly, the electrochemical performance of Fe_7_S_8_/FeS_2_/NCNT electrode can be further improved in ether-based electrolyte. We also systematically investigated the ion diffusion kinetics, charge storage mechanism, and SEI composition of the Fe_7_S_8_/FeS_2_/NCNT electrodes in different electrolytes.

## Experimental Section

### Preparation of Fe/NCNT

A facile pyrolysis method was applied to prepare the Fe/NCNT precursor. Typically, 10 mL of deionized water and 250 mg dicyandiamide (DICY) were mixed together under stirring, and then 50 mg ferric chloride (FeCl_3_) was added into the above aqueous solution and stirred for 1 h at room temperature. The mixture was then dried in 80 °C water bath to obtain light red powder (DICY-Fe). After that, the product was ground and calcined at 800 °C for 2 h under argon to receive the desired Fe/NCNT.

### Preparation of Fe_7_S_8_/FeS_2_/NCNT

The obtained Fe/NCNT and sulfur powder with a mass ratio of 1:3 were placed in an alumina boat, then annealed at 600 °C (heating rate of 2 °C min^−1^) for 2 h under argon atmosphere to get the Fe_7_S_8_/NCNT. Then the Fe_7_S_8_/NCNT and sulfur powder with a mass ratio of 1:1 were calcinated at 400 °C (heating rate of 2 °C min^−1^) for 1 h under argon to obtain the Fe_7_S_8_/FeS_2_/NCNT. For comparison, the Fe_7_S_8_/NCNT and sulfur powder with a mass ratio of 1:3 were calcinated at 400 °C for 4 h under argon to obtain the FeS_2_/NCNT.

The detailed material characterizations and electrochemical measurements were provided in the Supporting Information.

## Results and Discussion

### Physical Characterization Results

**Fig. 1:**
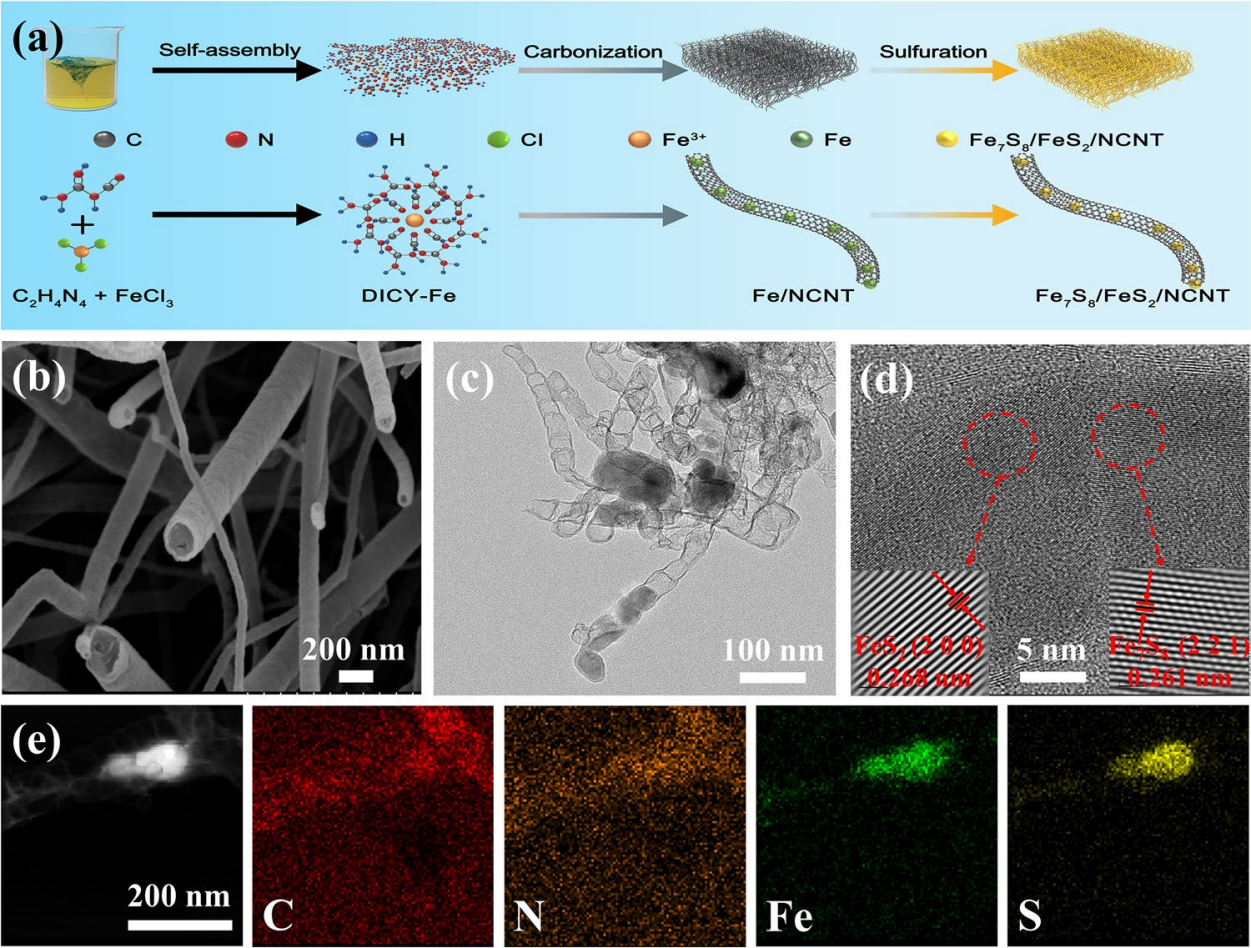
(**a**) Schematic illustration of the preparation, (**b**) SEM image, (**c**) TEM image, (**d**) HRTEM image, (**e**) STEM and corresponding elemental mapping images of Fe_7_S_8_/FeS_2_/NCNT

The fabrication strategy of Fe_7_S_8_/FeS_2_/NCNT is illustrated in Fig. 1a. Firstly, based on the coordination of the cyanogen groups in dicyandiamide (DICY) with the Fe^3+^, the unique DICY-Fe precursors can be obtained. Interestingly, owing to the high catalytic effect of iron species in the precursor, the nitrogen-doped carbon nanotubes can be formed during the subsequent carbonization process, resulting in the Fe/NCNT composite [31]. The subsequent sulfidation procedure in situ converts the Fe/NCNT into Fe_7_S_8_/FeS_2_ heterostructures coated by NCNT. The Fe_7_S_8_/FeS_2_ heterostructures can induce a large number of lattice defects and rapid charge transfer, which together with the excellent electronic conductivity and sufficient buffer space offered by the NCNT, contribute to boosting the electrochemical sodium storage performance [32, 33].

Scanning electron microscopy (SEM) and transmission electron microscopy (TEM) were employed to investigate the morphology features of the as-prepared samples. The SEM image of Fe/NCNT (Fig. S1a) reveals regular nanotube morphology with diameters of about tens of nanometers, indicating the conversion from the DICY-Fe complexes to Fe species confined into the carbon nanotubes after carbonization. The SEM images of Fe_7_S_8_/NCNT, FeS_2_/NCNT, and Fe_7_S_8_/FeS_2_/NCNT are shown in Figs. S1b, c and 1b, respectively. Their morphologies are not very different, and the nanotube morphologies are well maintained, indicating that the sulfidation treatment has little effect on their morphologies. The carbon nanotube possesses a highly conductive network to accelerate the charge transfer, the large specific surface area provides efficient buffer matrix, and the large internal space can accommodate volume expansion during repeated charging/discharging processes, all of which contribute to high sodium storage performance [34, 35]. Furthermore, TEM images of Fe_7_S_8_/FeS_2_/NCNT (Fig. 1c) demonstrate that the Fe_7_S_8_/FeS_2_ was embedded in the hollow carbon nanotube, which would be beneficial to alleviate the volume expansion of the Fe_7_S_8_/FeS_2_/NCNT heterostructure during electrochemical cycling process, thus maintaining the integrity of the electrode [34]. More structure information of the Fe_7_S_8_/FeS_2_/NCNT was obtained through the high-resolution transmission electron microscopy (HRTEM), where the lattice fringes with d-spacings of 0.261 and 0.268 nm correspond to (2 2 1) plane of Fe_7_S_8_ and (2 0 0) planes of FeS_2_, respectively, can be detected (Fig. 1d) [36, 37]. And lattice peak position period diagrams (Fig. S2) further prove the reliability of lattice spacings of Fe_7_S_8_ and FeS_2_. Moreover, the heterointerface between the Fe_7_S_8_ and FeS_2_ can be clearly observed, further indicating the successful synthesis of the heterostructures, which contribute to rapid charge transfer [33, 38]. Besides, the selected area electron diffraction (SAED) pattern of Fe_7_S_8_/FeS_2_/NCNT (Fig. S3) manifests that the clear diffraction rings can be well indexed to the (4 1 1), and (2 2 0) planes of cubic FeS_2_ and (− 2 2 1) and (− 6 2 3) planes of monoclinic Fe_7_S_8_ [36, 37]. The STEM and related elemental mappings of Fe_7_S_8_/FeS_2_/NCNT are displayed in Fig. 1e. Clearly, C and N elements are uniformly distributed throughout the structure, while Fe and S are concentrated in the lumen of the nanotube, further showing the confined structure [38]. The intercalation design of heterostructure in NCNTs can significantly alleviate the structure expansion/contraction of the electrode and effectively improve the electronic conductivity of the composite [38].

**Fig. 2:**
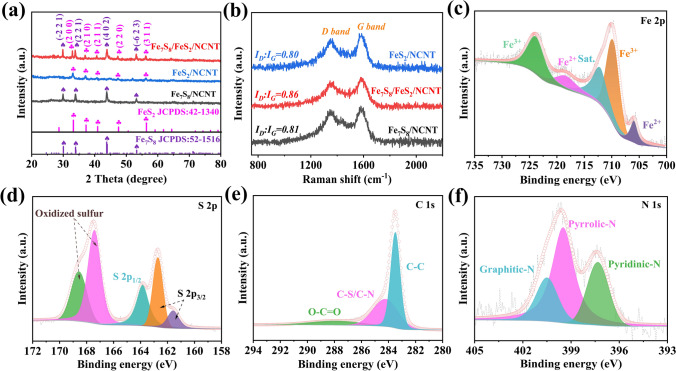
(**a**) XRD patterns and (**b**) Raman spectra of the Fe_7_S_8_/NCNT, FeS_2_/NCNT, and Fe_7_S_8_/FeS_2_/NCNT. XPS spectra of the Fe_7_S_8_/FeS_2_/NCNT: (**c**) Fe 2*p*, (**d**) S 2*p*, (**e**) C 1* s* and (**f**) N 1* s*

To preliminarily determine the phase structures of as-prepared samples, X-ray powder diffraction (XRD) measurements were performed. Particularly, the diffraction peaks of the as-synthesized Fe/NCNT (Fig. S4) can be well indexed to the cubic Fe (JCPDS No. 03–1050), indicating the successful conversion from the DICY-Fe to Fe/NCNT [39]. In Fig. 2a, the XRD pattern of Fe_7_S_8_/FeS_2_/NCNT manifests the characteristic peaks of monoclinic Fe_7_S_8_ (JCPDS No. 52–1516) and cubic FeS_2_ (JCPDS No. 42–1340) [40, 41]. Specifically, the diffraction peaks located at around 29.9°, 33.8°, 43.7°, and 53.2° correspond to the (− 2 1 1), (2 2 1), (4 0 2), and (− 6 2 3) planes of monoclinic Fe_7_S_8_, while the diffraction peaks centered at around 33.1°, 37.1°, 40.7°, 47.4° and 56.3° can be ascribed to the (2 0 0), (2 1 0), (2 1 1), (2 2 0) and (3 1 1) planes of cubic FeS_2_, respectively, thereby indicating the successful fabrication of the target heterostructure [40, 41]. For comparison, the XRD patterns of Fe_7_S_8_/NCNT and FeS_2_/NCNT can be identified with monoclinic Fe_7_S_8_ and cubic FeS_2_, respectively. While no diffraction peak related to the NCNT can be observed, mainly due to their amorphous state and low content. To confirm the existence of NCNT, Raman spectra are performed (Fig. 2b). Apparently, two broad peaks can be detected at around 1588 and 1351 cm^−1^, which is attributed to the graphitic structure (G bands) and disordered domain (D bands) in the carbonaceous skeleton, respectively [27]. Particularly, the higher* I*_D_/*I*_G_ value of Fe_7_S_8_/FeS_2_/NCNT (0.86) than those of Fe_7_S_8_/NCNT (0.81) and FeS_2_/NCNT (0.80) means that the more defects generated in the heterostructure, offering extra active sites and contributing to improving the sodium storage performance [29]. Meanwhile, the NCNT content of Fe_7_S_8_/FeS_2_/NCNT is characterized by thermogravimetric (TG) analysis. As shown in Fig. S5, the weight loss is attributed to the oxidation of Fe_7_S_8_/FeS_2_ and N-doped carbon in air. Based on the fact that the sulfidation process will not change the mass ratio of Fe element to NCNT from Fe/NCNT to Fe_7_S_8_/FeS_2_/NCNT, thus the content of NCNT in Fe_7_S_8_/FeS_2_/NCNT is calculated to be 30.4% [16].

Furthermore, the surface component and elemental oxidation state of the obtained Fe_7_S_8_/NCNT, Fe_7_S_8_/FeS_2_/NCNT, and FeS_2_/NCNT samples were characterized through X-ray photoelectron spectroscopy (XPS). The XPS survey spectra of all samples shown in Fig. S6 verify the co-existence of elements Fe, S, C, and N in the target samples. Note that the appearance of O 1*s* peak should be ascribed to the exposure to air [18]. As shown in Fig. 2c, the Fe 2*p* spectrum of Fe_7_S_8_/FeS_2_/NCNT exhibits two peaks at 706.2 and 718.6 eV, respectively, which confirms the existence of Fe^2+^, and the characteristic signals for Fe^3+^ can be observed at around 710.6 and 724.6 eV [19]. For the S 2*p* spectrum shown in Fig. 2d, the S 2*p*_3/2_ species can be detected at around 161.6 and 162.7 eV. In addition, the presence of S 2*p*_1/2_ can be confirmed by the peak detected at 163.8 eV, and two peaks at around 167.5 and 168.6 eV correspond to oxidized sulfur, which may be attributed to the oxidization of metal sulfides in air [27]. Regarding the N 1*s* spectrum (Fig. 2e), three peaks located at around 397.3 (pyridinic-N), 399.5 (pyrrolic-N), and 400.5 (graphitic-N) eV, respectively, can be detected [42]. In regard to the C 1*s* spectrum (Fig. 2f), a dominant peak at around 283.5 eV can be indexed to the C–C, while another two weak peaks at 284.0 and 288.1 eV can be ascribed to the C–N/C–S and O–C = O, respectively [43]. For comparation, the XPS spectra of the Fe_7_S_8_/NCNT and FeS_2_/NCNT were also investigated, as shown in Figs. S7 and S8. Both the N 1*s* and C 1*s* spectra of the Fe_7_S_8_/NCNT and FeS_2_/NCNT composites are almost identical to those of Fe_7_S_8_/FeS_2_/NCNT. Apparently, the Fe 2*p* and S 2*p* spectra of Fe_7_S_8_/NCNT and FeS_2_/NCNT show distinct differences, revealing different valence states of Fe and S in the two samples. Notably, the obvious oxidized sulfur peak in the S 2*p* spectrum of FeS_2_/NCNT should be ascribed to the surface oxidization upon sulfidation [27].

### Electrochemical Results

**Fig. 3:**
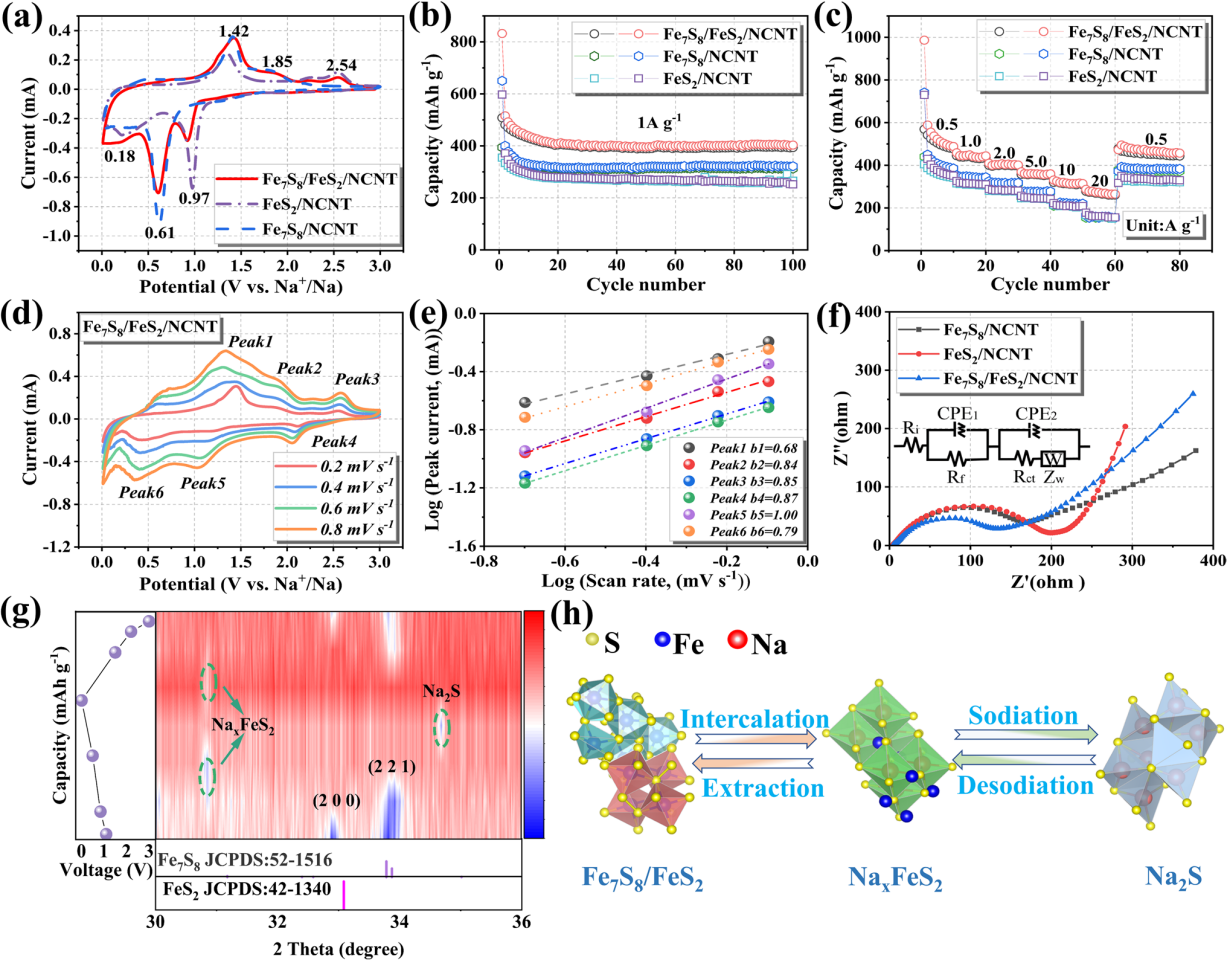
(**a**) Comparison of the first CV curves at 0.2 mV s^−1^, (**b**) cycling performances, and (**c**) rate capabilities of Fe_7_S_8_/NCNT, FeS_2_/NCNT, and Fe_7_S_8_/FeS_2_/NCNT electrodes. (**d**) CV curves at different scan rates (from 0.2 to 0.8 mV s^−1^) and (**e**) linear relationship of peak currents Log (i) versus scan rate Log (v) of the Fe_7_S_8_/FeS_2_/NCNT electrode. (**f**) Nyquist plots of the Fe_7_S_8_/NCNT, FeS_2_/NCNT, and Fe_7_S_8_/FeS_2_/NCNT electrodes after 50 cycles at 1.0 A g^−1^. (**g**) XRD contour map at different charging/discharging states, and (**h**) reaction mechanism illustration during sodiation and desodiation process of the Fe_7_S_8_/FeS_2_/NCNT electrode

The sodium storage performances of as-prepared samples were investigated in ester-based electrolyte. The comparison of initial cyclic voltammetry (CV) curves of Fe_7_S_8_/NCNT, Fe_7_S_8_/FeS_2_/NCNT, and FeS_2_/NCNT electrodes are shown in Fig. 3a. Compared with the initial CV curves of Fe_7_S_8_/NCNT and FeS_2_/NCNT electrodes, the Fe_7_S_8_/FeS_2_/NCNT exhibits reduction peaks at 0.97 and 0.61 V, which can be assigned to the Na^+^ insertion into cubic FeS_2_ and monoclinic Fe_7_S_8_, respectively [18]. Subsequently, the reduction peak at 0.18 V can be indexed to the conversion from Fe^2+^/Fe^3+^ to Fe^0^ [18]. As for the subsequent anodic scan, the formation of Na_x_FeS_2_ can be confirmed through the peak at around 1.42 V, while another two peaks at around 1.85 and 2.54 V can be ascribed to the desodiation of Na_x_FeS_2_ [37, 41]. To detect the detailed electrochemical behaviors of the obtained Fe_7_S_8_/FeS_2_/NCNT, the initial four CV curves at 0.2 mV s^−1^ have been depicted in Fig. S9a. The irreversible phase transition at the first cathodic scan can be attributed to the passivation process of the electrode surface, accompanied by the decomposition of electrolyte and the generation of SEI [44]. Afterward, the substantially overlapping CV curves of the Fe_7_S_8_/FeS_2_/NCNT indicate its excellent electrochemical reversibility and structural stability [44]. In contrast, the Fe_7_S_8_/NCNT and FeS_2_/NCNT electrodes show reduced electrochemical reversibility, which can be detected from Fig. S9b, c. Accordingly, the voltage plateaus in the charge/discharge voltage curves of Fe_7_S_8_/FeS_2_/NCNT, Fe_7_S_8_/NCNT, and FeS_2_/NCNT electrodes are highly consistent with the redox peaks presented in the CV results (Fig. S9d–f).

To further highlight the structural advantage of the Fe_7_S_8_/FeS_2_/NCNT as anode material for SIBs, the comparison of cycling stability of the as-fabricated electrodes was conducted (Fig. 3b). The Fe_7_S_8_/FeS_2_/NCNT, Fe_7_S_8_/NCNT, and FeS_2_/NCNT electrodes deliver reversible capacities of 403.2, 321.7 and 252.1 mAh g^−1^, respectively, at 1.0 A g^−1^ after 100 cycles, indicating the excellent electrochemical activity of the designed heterostructure. In regard to the rate capability, the Fe_7_S_8_/FeS_2_/NCNT electrode can deliver average reversible capacities of 521.9 (0.5 A g^−1^), 448.5 (1.0 A g^−1^), 406.6 (2.0 A g^−1^), 359.7 (5.0 A g^−1^) and 316.7 (10.0 A g^−1^) mAh g^−1^, respectively (Fig. 3c). Surprisingly, a satisfied reversible capacity of 273.4 mAh g^−1^ can still be achieved even at a high current density of 20.0 A g^−1^, which means that the battery can be fully charged and discharged within 1 min. In contrast, the Fe_7_S_8_/NCNT and FeS_2_/NCNT electrodes can only deliver reversible capacities of 153.8 and 161.1 mAh g^−1^ at 20.0 A g^−1^, respectively. Besides, the charge–discharge rate profiles of the Fe_7_S_8_/FeS_2_/NCNT electrode reveal that the voltage plateaus of the curves at different current densities can be well maintained (Fig. S10a). For the Fe_7_S_8_/NCNT and FeS_2_/NCNT electrodes, the gradually degraded plateaus and decreased capacities suggest their poor structure stability (Fig. S10b, c) [45].

In order to reveal the kinetics origin and reaction mechanism of the target Fe_7_S_8_/FeS_2_/NCNT electrode, CV curves collected from 0.2 to 0.8 mV s^−1^ scan rates have been performed (Fig. 3d). Compared with the CV curves of Fe_7_S_8_/NCNT and FeS_2_/NCNT electrode (Fig. S11), the CV shape of Fe_7_S_8_/FeS_2_/NCNT is well-maintained with increasing scan rate, indicating its weak electrochemical polarization and excellent electrochemical reversibility. It is well known that both diffusion and capacitive behaviors contribute to the charge stored in electrode, and the response current and scan rate obey the following equation [12, 45]:

1 $${\text{i = a v}}^{{\text{b }}}$$

where *a* is a variable parameter, and the value of *b* is a typical index to judge the reaction kinetics, and it can be received from the slope after plotting log (*i*) versus log (*v*). Generally, the value of *b* close to 1.0 corresponds to a pseudocapacitive process, while the value approaching to 0.5 means that the electrochemical process is dominated by diffusion [46]. In Fig. 3e, the *b* values after fitting for the oxidation peaks are 0.68 (peak 1), 0.84 (peak 2), and 0.85 (peak 3), and the corresponding values of the reduction peaks are 0.87 (peak 4), 1.00 (peak 5) and 0.79 (peak 6), indicating that the charge stored in the Fe_7_S_8_/FeS_2_/NCNT electrode is dominated by both diffusion and capacitive behaviors [13, 32]. Generally, the mixed charge storage mechanism can not only provide high reaction kinetics through the pseudocapacitive behavior, but also induce high utilization of the active material, thus guaranteeing high electrochemical sodium storage performance [32]. In addition, the Nyquist plots of the three electrodes after 50 cycles at 1.0 A g^−1^ manifest that the Fe_7_S_8_/FeS_2_/NCNT electrode displays a lower charge transfer resistance (93.5 Ω) compared with those of Fe_7_S_8_/NCNT (130.1 Ω) and FeS_2_/NCNT (134.4 Ω), indicating better electrochemical kinetics of the special designed Fe_7_S_8_/FeS_2_/NCNT heterostructure (Fig. 3f) [21, 23]. Therefore, the high capacitive contribution of the charge storage as well as the low energy barrier of charge transfer can facilitate the electrons/ions transport, thus dramatically decreasing the electrochemical polarization.

Furthermore, in order to reveal the working mechanism of the Fe_7_S_8_/FeS_2_/NCNT electrode, ex situ XRD measurements for the target electrode at selected voltages upon sodiation/desodiation were performed. The cells under the selected voltages were disassembled for ex situ XRD tests (Fig. S12), and the corresponding contour plot in the range of 30° to 36° can observe the phase change more clearly (Fig. 3g) [47–49]. The diffraction peaks of the monoclinic Fe_7_S_8_ and cubic FeS_2_ can be obviously observed in the pristine electrode. After discharging to 0.75 V, the diffraction peak of the FeS_2_ disappears and the Na_x_FeS_2_ phase gradually forms, which can be attributed to the intercalation of Na^+^ into the FeS_2_ [21, 22]. After further discharging to 0.48 V, the diffraction peaks of Fe_7_S_8_ and FeS_2_ disappear completely. Subsequently, diffraction peaks of NaS_2_ and Fe can be observed in the fully discharged state (0.05 V), suggesting that Fe_7_S_8_/FeS_2_/NCNT experiences the Na^+^ insertion and phase transition upon discharge [18]. During the charging process, the characteristic peaks, corresponding to the Na_2_S and Fe, gradually weaken, combined with the appearance of Na_x_FeS_2_, suggesting the deintercalation of Na^+^. Finally, the formation of FeS_2_ and Fe_7_S_8_ suggests the reverse phase transition, highlighting its superior electrochemical reversibility [23]. Figure 3h typically illustrates the working mechanism of Fe_7_S_8_/FeS_2_/NCNT electrode during sodiation/desodiation process. It undergoes a two-step reaction mechanism upon sodium storage: the Na^+^ intercalation and phase transformation during the discharge process; the reverse conversion reaction and Na^+^ extraction during the charging process [23].

**Fig. 4:**
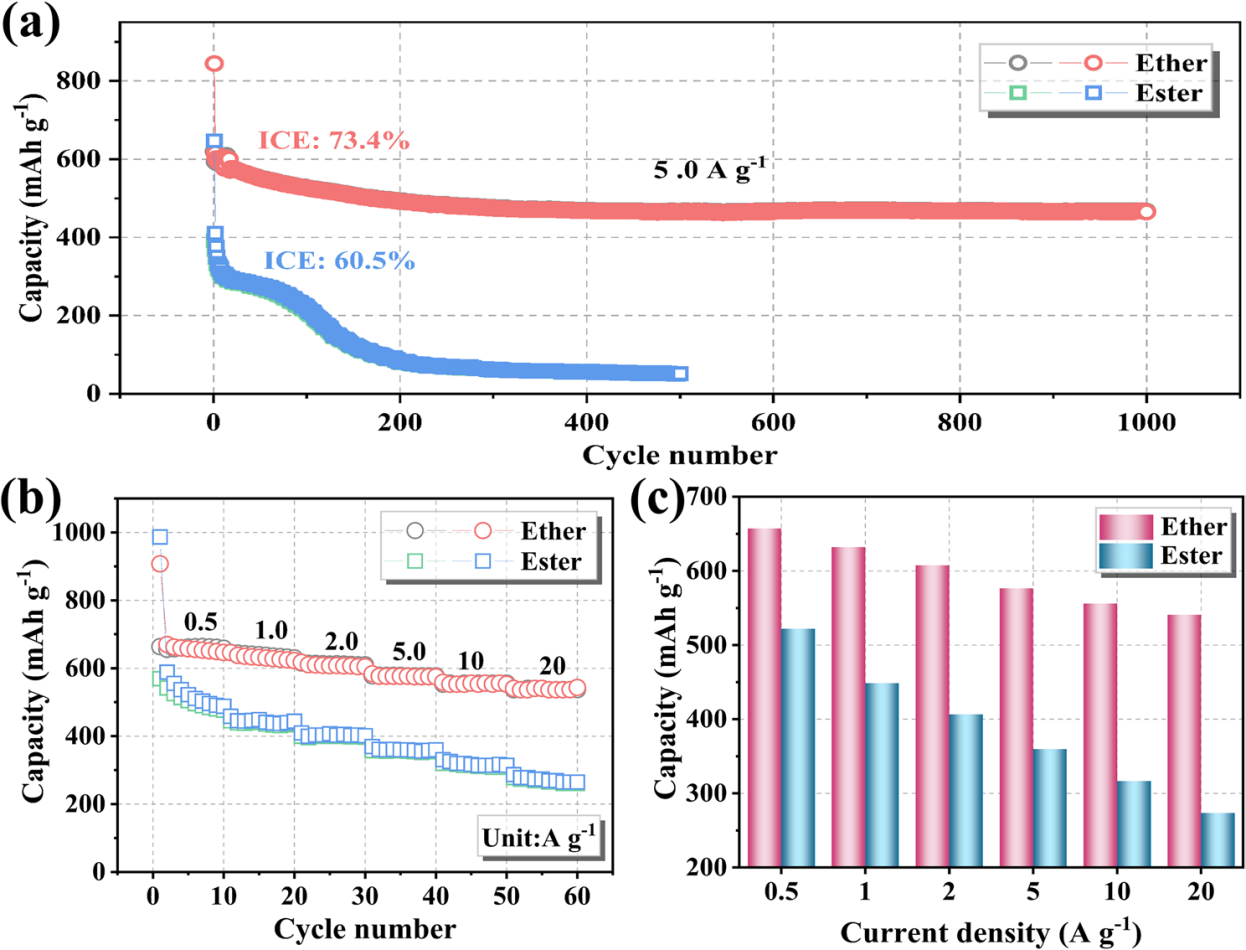
(**a**) Cycling performance and (**b**) rate performance comparison of Fe_7_S_8_/FeS_2_/NCNT electrode in ester/ether-based electrolytes; (**c**) corresponding specific capacity at various current densities in two different electrolytes

In addition to improving electrochemical performance from material synthesis perspective, it has been found that electrolytes can greatly influence the structural evolution and electrochemical performance of iron sulfide-based electrodes in SIBs. Figure 4 compares the cycling performance and rate capacity of Fe_7_S_8_/FeS_2_/NCNT electrodes in ester/ether-based electrolytes. Figure 4a evaluates the cycling stability of the Fe_7_S_8_/FeS_2_/NCNT electrode in those two electrolytes. Particularly, an impressive capacity of 466.7 mAh g^−1^ is maintained after 1,000 cycles at 5.0 A g^−1^ in the ether-based electrolytes, demonstrating its robust cycle life. For comparison, negligible capacity can be detected of Fe_7_S_8_/FeS_2_/NCNT electrode after 500 cycles in the ester-based electrolyte. In addition, the initial Coulombic efficiency (ICE) of 73.4% can be obtained in the ether-based electrolyte, which is much higher than that in the ester-based electrolyte (60.5%). This could be attributed to the high electrochemical reversibility of electrode in ether-based electrolyte. Besides, a superior CE can be detected in ether-based electrolyte (~ 100%) than that in ester-based electrolyte (Fig. S13). In general, the excellent CE in the ether-based electrolyte could be attributed to the uniform and stable SEI generated on the electrode surfaces, which can efficiently prevent further consumption of electrolyte and thus improve electrochemical reversibility [30, 50].

Figure 4b-c further compares the rate capacities of Fe_7_S_8_/FeS_2_/NCNT electrodes in those two electrolytes. Even at 20 A g^−1^, a high capacity of 536.5 mAh g^−1^ can be maintained in the ether-based electrolyte, and the capacity retention rate is about 81.7% for the reversible capacity at 0.5 A g^−1^. However, only 52.4% of the capacity can be retained in the ester-based electrolyte. Figure S14 shows discharge–charge profiles of the Fe_7_S_8_/FeS_2_/NCNT electrode at different current densities in the ether-based electrolyte. The charge/discharge curves at different current densities are almost consistent and show longer charge potential plateaus, contributing to the outstanding rate capability and high specific capacity [51]. Besides, the superior electrochemical sodium storage performance of as-prepared Fe_7_S_8_/FeS_2_/NCNT can be also confirmed through the comparison with other related studies (Table S1), from where the target Fe_7_S_8_/FeS_2_/NCNT shows excellent sodium storage, mainly resulting from the heterostructure design and combination with NCNT [16].

**Fig. 5:**
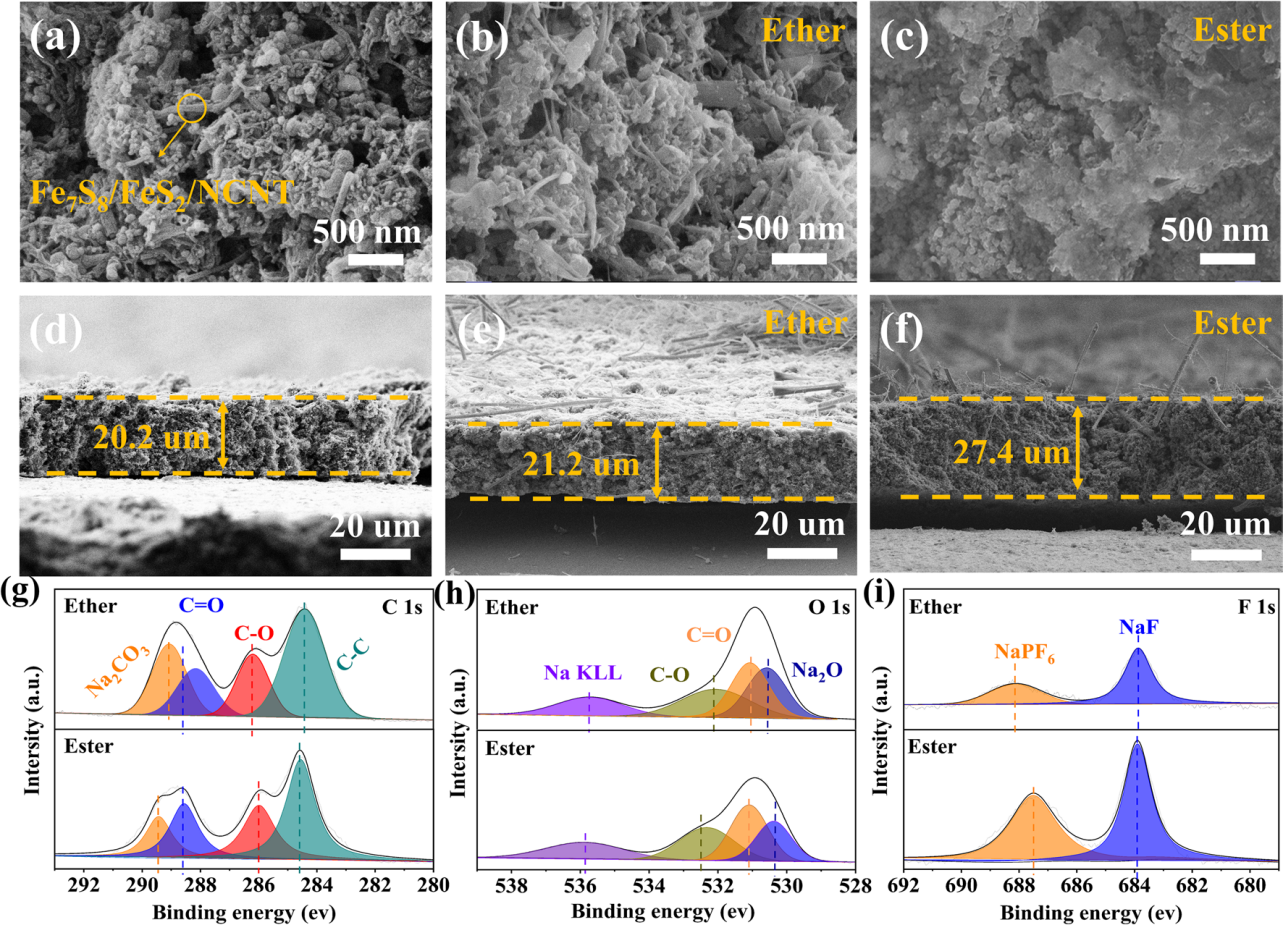
(**a**) SEM and (**d**) cross-sectional images of the pristine Fe_7_S_8_/FeS_2_/NCNT electrode. After 100 cycles at 1.0 Ag^−1^, the SEM and cross-sectional images in (**b**, **e**) ether and (**c**, **f**) ester-based electrolytes. Comparison of the XPS spectra of Fe_7_S_8_/FeS_2_/NCNT electrode after 100 cycles in these two electrolytes: (**g**) C 1s, (**h**) O 1s, (**i**) F 1s

Although the ether-based electrolyte has been identified as a key factor in achieving the excellent electrochemical properties of TMS-based anodes for SIBs, the origin of the positive effect of ether-based electrolyte on TMS-based anodes remains poorly understood. To prove the positive effect of ether-based electrolyte on electrode integrity, SEM investigations were performed on the electrode after cycling. The SEM image of the pristine Fe_7_S_8_/FeS_2_/NCNT electrode shows the typical tubular structure, which indicates that the electrode fabrication process plays little effect on its morphology (Fig. 5a). After cycling, the microstructure of Fe_7_S_8_/FeS_2_/NCNT electrode is well maintained in the ether-based electrolyte, and the tubular morphology is still clearly visible, which highlights the robust integrity of the electrode in ether-based electrolyte (Fig. 5b). In contrast, obvious agglomeration on the electrode after cycling can be detected in ester-based electrolyte (Fig. 5c), indicating the large volume change upon cycling [50, 52]. Furthermore, the cross-sectional SEM images show that the thickness of the pristine electrode and the thickness of the electrodes after cycling in ether-based and ester-based electrolytes are 20.2, 21.2, and 27.4 μm, respectively (Fig. 5d, e, f). The swelling ratio of the electrode in ester-based electrolyte is 35.6%, which is much higher than that in the ether-based electrolyte (5%), demonstrating that the structural change of the electrode is alleviated after coupling with ether-based electrolyte [52, 53].

It's well known that ether-based electrode can induce a dense and robust SEI film on electrode surface, thereby providing fast charge transfer at the interface, reducing irreversible reactions, and maintaining the electrode integrity [52, 53]. To observe the chemical components on the SEI layer formed in different electrolytes, XPS measurements were carried out on the electrode after cycling. In the C 1*s* spectra of the electrodes, the characteristic peaks corresponding to C = O, Na_2_CO_3_, C–O, and C–C can be found in both two electrolytes (Fig. 5g). Meanwhile, the O 1*s* spectra exhibit four fitting peaks corresponding to the Na_2_O, C–O, C = O, and polyester, respectively (Fig. 5h) [13, 27]. Clearly, the Na_2_CO_3_ and Na_2_O signals account for a larger proportion in the ether-derived SEI layer, which means that the ether-derived SEI film is mainly composed of inorganic components. It has been reported that relatively high inorganic components in the SEI layer are more likely to form a robust and dense SEI film, contributing to interfacial kinetics and charge transfer [52, 53]. Regarding the F 1*s* spectra, signals ascribed to the NaF and NaPF_6_ species can be detected in both electrolytes (Fig. 5i) [53]. While the peak areas of NaF and NaPF_6_ in ether-derived SEI layer are much smaller than those of ester-derived SEI layer, indicating a large amount of electrolyte is consumed during the SEI generation in the ester-based electrolytes. This further proves that the electrode is more stable when cycled in the ether-based electrolyte [50, 54].

**Fig. 6:**
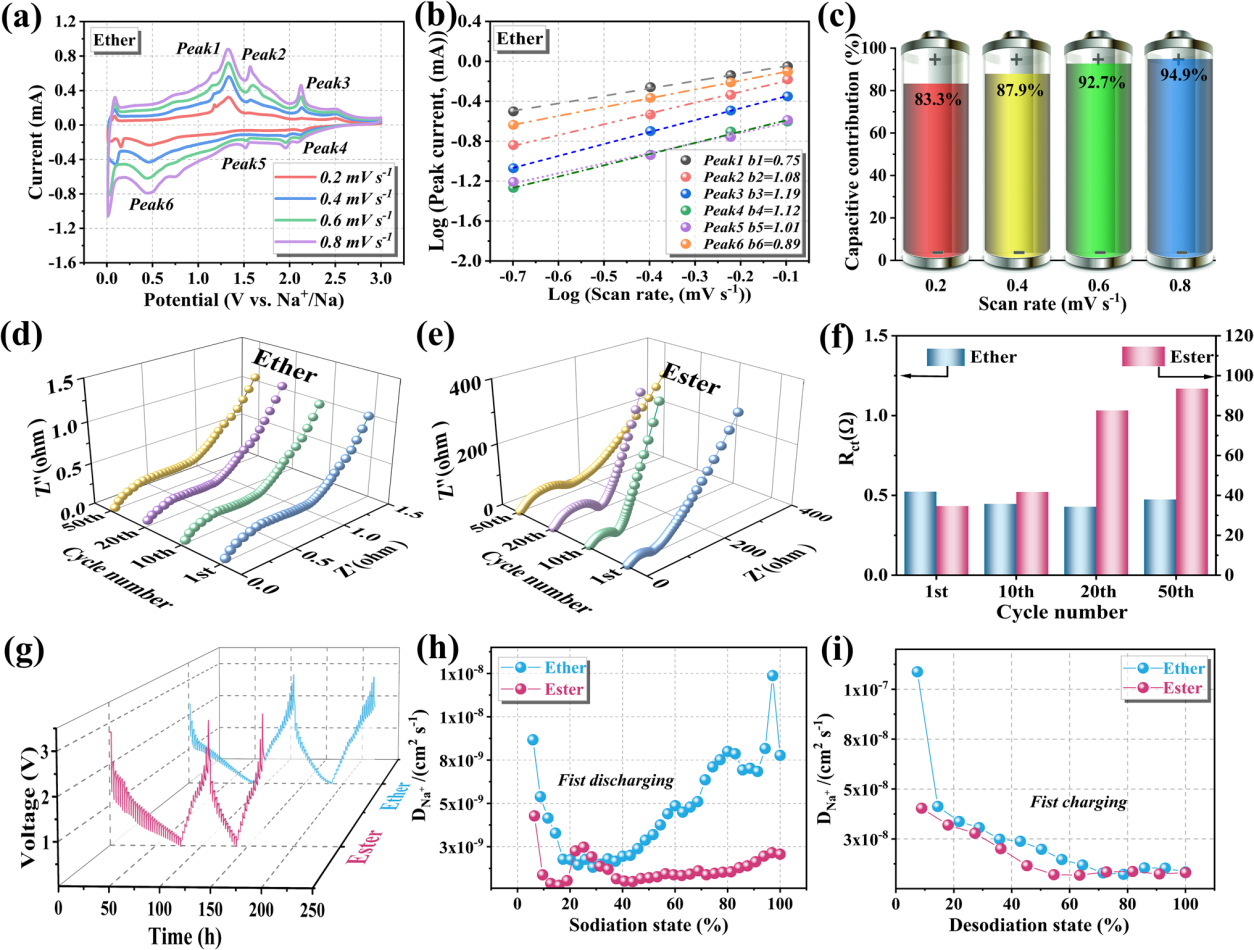
(**a**) The CV curves at different scan rates (from 0.2 to 0.8 mV s^−1^), (**b**) linear relationship of peak currents Log (i) versus scan rate Log (v), (**c**) capacity contributions of capacitive and diffusion-controlled behaviors at different scan rates of the Fe_7_S_8_/FeS_2_/NCNT electrode in the ether-based electrolyte. Nyquist plots of the Fe_7_S_8_/FeS_2_/NCNT electrode at 1st, 10th, 20th, and 50th cycles at 1 A g^−1^ from 0.01 to 10^7^ Hz in (**d**) ether-based and (**e**) ester-based electrolytes, and (**f**) corresponding *R*_ct_ values. (**g**) GITT curves of the Fe_7_S_8_/FeS_2_/NCNT electrode during the first two cycles as a function of time in the voltage of 0.01 to 3 V in different electrolytes. The diffusion coefficients of sodium ion during the initial (**h**) discharge and (**i**) charge process in different electrolytes

Next, CV, electrochemical impedence spectroscopy (EIS), and galvanostatic intermittent titration (GITT) were conducted to evaluate Na^+^ kinetics differences of Fe_7_S_8_/FeS_2_/NCNT electrode in the ester/ether-based electrolytes. First of all, the electrochemical behaviors of the Fe_7_S_8_/FeS_2_/NCNT in the ether-based electrolyte (0.01–3 V) have been investigated through the CV measurements (Fig. 6a), which match well with that in the ester-based electrolyte. Moreover, compared with the cathodic/anodic peaks in the ester-based electrolyte, the redox peaks become much sharper in the ether-based electrolyte, indicating an improved redox activity. Additionally, the CV curves of Fe_7_S_8_/FeS_2_/NCNT electrode at various scan rates in the ether-based electrolyte display significant left shifting of the cathodic peak and right shifting of the anodic peak, demonstrating its faintish electrochemical polarization [50]. Meanwhile, the charge/discharge profiles of the Fe_7_S_8_/FeS_2_/NCNT electrode exhibit increased discharge platform and reduced charge platform, which indicate lower electrochemical polarization in the ether-based electrolyte, leading to the superior redox reversibility (Fig. S15) [54, 55]. Furthermore, the calculated b values for the Fe_7_S_8_/FeS_2_/NCNT in ether-based electrolyte are higher than those in ester-based electrolyte, which indicate that the electrode coupled with ether-based electrolyte features rapid interfacial kinetics and larger capacitive contribution for charge storage (Fig. 6b) [30, 32]. To be more specific, the capacitive contribution at various scan rates can be verified by the following equation [56, 57]:

2 $${\text{i = k}}_{{1}} {\text{v + k}}_{{2}} {\text{v}}^{{1/2}}$$

where k_1_v represents the contribution from capacitive behaviors, while k_2_v^1/2^ corresponds to the contribution from diffusion [56, 57]. As can be seen from Fig. 6c, the capacitive contributions in ether-based electrolyte gradually increase from 83.3% to 94.9% as the scan rate increases, which are much larger than those in the ester-based electrolyte (Fig. S16). Specifically, after data collection and fitting, as displayed by the shaded areas in Figs. S17 and S18. Generally, good electrochemical kinetics at high current densities can be obtained from high capacitive contribution, which is beneficial for the rate capability and cycling stability [57].

The EIS comparison of the Fe_7_S_8_/FeS_2_/NCNT electrode at 1st, 10th, 20th, and 50th cycles at 0.1 A g^−1^ in ether/ester-based electrolytes have been illustrated in Fig. 6d, e. The semicircle and straight line observed at the high-frequency and low-frequency regions represent the charge transfer resistance (*R*_ct_) and Warburg factor, respectively [58]. As shown in Fig. 6f, the lower *R*_ct_ values in ether-based electrolyte indicate rapid charge transfer and stable interface [56]. For the ester-based electrolyte, the *R*_ct_ value increases significantly from 34.6 (1st cycle) to 93.5 Ω (50th cycle), which can be attributed to the formation of a thick and unstable SEI layer caused by the ester-based electrolyte and then slow down the interfacial kinetics [53].

Besides, GITT investigation was carried out to compare the Na^+^ diffusion coefficient in different electrolytes. In general, the diffusion coefficient of sodium ion can be evaluated based on the following equation [59, 60]:

3 $$D_{{Na^{ + } }} = \frac{4}{\pi \tau }\left( {\frac{{{\text{m}}_{{\text{b}}} {\text{V}}_{{\text{m}}} }}{{{\text{M}}_{{\text{b}}} {\text{S}}}}} \right)^{2} \left( {\frac{{\Delta {\text{E}}_{{\text{S}}} }}{{\Delta {\text{E}}_{{\uptau }} }}} \right)^{2} ,\;\tau \ll L^{2} /{\text{D}}_{{{\text{Na}}^{ + } }}$$

where *M*_b_, *m*_b_, *V*_m_, *S* and τ represent the molecular weight, the mass loading of active material, the molar volume of the electrode material, the surface area of the electrode, and the applied current, respectively, while Δ*E*_s_ and Δ*E*_τ_ correspond to the steady-state voltage change after the discharge pulse and the voltage variation during the discharge pulse [59, 60]. Figure 6g–i depicts the GITT curves and the calculated diffusion coefficients of the Fe_7_S_8_/FeS_2_/NCNT electrode in ether-based and ester-based electrolytes (mainly ranging from 10^−11^ to 10^−7^ cm^2^ s^−1^). Specifically, the average diffusion coefficients of Na^+^ upon the first discharge and charge in the ether-based electrolyte are 4.71 × 10^−9^ and 2.66 × 10^−8^ cm^2^ s^−1^, respectively. As a comparison, the ester-based electrolyte exhibits relatively low diffusion coefficients, obtaining 1.24 × 10^−9^ and 1.62 × 10^−8^ cm^2^ s^−1^ during the first discharge and charge, respectively. Similarly, the variation trend of Na^+^ diffusion coefficients in the second cycle of the two electrolyte is similar to that in the first cycle (Fig. S19), suggesting good electrochemical reversibility and stable ion transport channels [59, 60].

## Conclusions

In summary, the rationally designed Fe_7_S_8_/FeS_2_/NCNT heterostructures have been successfully fabricated via an in situ pyrolysis and sulfidation strategy. The as-prepared Fe_7_S_8_/FeS_2_/NCNT features Fe_7_S_8_/FeS_2_ heterostructure embedded in the hollow carbon nanotubes. The unique nano-architecture provides abundant lattice defects, superior electronic conductivity, and sufficient buffer space to maintain the integrity of the electrode during cycling. Consequently, the Fe_7_S_8_/FeS_2_/NCNT electrode delivered high sodium storage capacity, rapid ionic diffusion kinetics, and remarkable electrochemical reversibility. In particular, the Fe_7_S_8_/FeS_2_/NCNT electrode exhibits even more stable long-term cycling performance (466.7 mAh g^−1^ after 1,000 cycles at 5 A g^−1^) and rate capability (536.5 mAh g^−1^ at 20 A g^−1^) in ether-based electrolyte, which endows the battery with rapid ion diffusion kinetics, low charge-transfer resistance, high capacitive contribution to charge storage and robust SEI layer. The advanced Fe_7_S_8_/FeS_2_/NCNT heterostructure design and in-depth investigation on the origin of the superior electrochemical performances in ether-based electrolyte have paved the way for the development of TMS-based electrodes in sodium storage system.

### Supplementary Information

Below is the link to the electronic supplementary material.

Supplementary file1 (PDF 2050 kb)
