# Supplementary material for: Interface Engineering of Fe7S8/FeS2 Heterostructure in situ Encapsulated into Nitrogen-Doped Carbon Nanotubes for High Power Sodium-Ion Batteries
Source: Nanomicro Lett. 2023 Apr 30;15:118. doi: 10.1007/s40820-023-01082-w (PMC10149539; doi:10.1007/s40820-023-01082-w)
Supplement: Supplementary file 1 — Supplementary file1 (PDF 2050 kb) [file 40820_2023_1082_MOESM1_ESM.pdf]

Supporting Information for

Interface Engineering of Fe_7S_8/FeS_2 Heterostructure *in-situ* Encapsulated into Nitrogen-Doped Carbon Nanotubes for High Power Sodium-Ion Batteries

Penghao Song[1], Jian Yang[1, 3], Chengyin Wang[1], Tianyi Wang[1, *], Hong Gao[2, *], Guoxiu Wang[2, *], Jiabao Li[1, *]

[1]College of Chemistry and Chemical Engineering, Yangzhou University, 180 Si-Wang-Ting Road, Yangzhou, Jiangsu 225002, P. R. China

[2]Centre for Clean Energy Technology, Faculty of Science, University of Technology Sydney, PO Box 123, Broadway, NSW 2007, Australia

[3]Key Lab of Fluorine and Silicon for Energy Materials and Chemistry of Ministry of Education, College of Chemistry and Chemical Engineering, Jiangxi Normal University, Nanchang 330022, P. R. China

*Corresponding authors. E-mail: wangty@yzu.edu.cn (Tianyi Wang); hong.gao@uts.edu.au (Hong Gao); guoxiu.wang@uts.edu.au (Guoxiu Wang); jiabaoli@yzu.edu.cn (Jiabao Li)

S1 Material Characterization

The phase structure of the as-prepared samples was investigated through X-ray powder diffraction (XRD, Holland Panalytical PRO PW 3040/60). Raman spectra were tested on RM-1000 (RENISHAW). The surface chemical composition was performed by X-ray photoelectron spectroscopy (XPS, Thermo ESCALAB 250 XI). The morphology and microstructure of the as-prepared samples were investigated through field-emission scanning electron microscopy (FESEM, Hitachi-4800), transmission electron microscopy (TEM), and high-resolution transmission electron microscopy (HRTEM, JEOL, JEM-2010).

S2 Electrochemical Test

Firstly, the as-prepared samples (Fe_7S_8/NCNT, Fe_7S_8/FeS_2/NCNT, and FeS_2/NCNT) were mixed with Super P and carboxymethylcellulose sodium in deionized water at a weight ratio of 7:2:1, respectively, and stirred to obtain a uniform slurry. Then the slurry was coated on copper foil, vacuum-dried at 60 °C overnight, and cut into 12 mm diameter disks to obtain the working electrodes. The average mass loading of the active material is around 1.2×10^{-3} g cm^{-2}. Then assembling the half cells in an argon-filled glovebox, with sodium foil and Whatman glass fiber as the reference electrode and

separator, respectively. Ester-based electrolyte (1.0 M of $NaPF_6$ in ethylene carbonate and diethyl carbonate (EC/ DEC = 1/1 by volume)) and ether-based electrolyte (1 M $NaPF_6$ in diglyme (DME)) were employed. The galvanostatic cycle tests of half cells were conducted in the NEWARE battery test system with a wide voltage window of 0.01-3 V. To further investigate the Na^+ storage kinetics, cyclic voltammetry (CV) curves were obtained using an electrochemical workstation (CHI 660D) at different scan rates. The electrochemical impedance spectroscopy (EIS) measurements were carried out on the same electrochemical workstation (CHI 660D).

S3 Supplementary Figures and Table

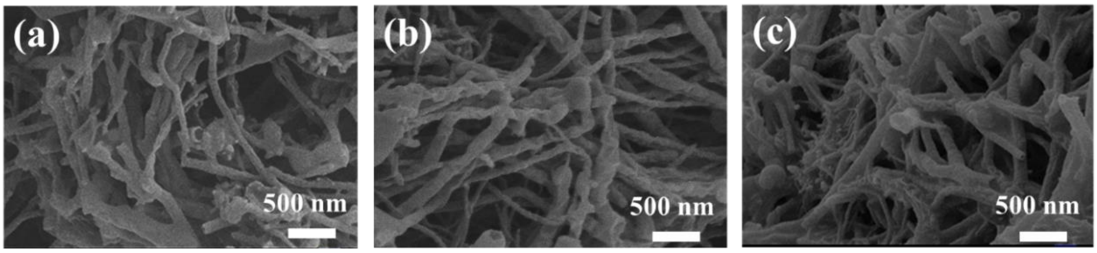

Fig. S1 SEM images of **a** Fe/NCNT, **b** Fe_7S_8/NCNT, and **c** FeS_2/NCNT

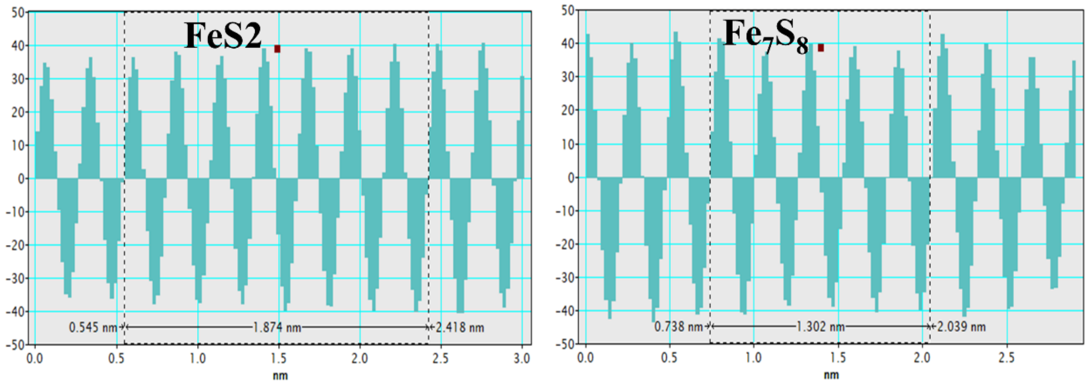

Fig. S2 Crystal plane spacing periodogram of Fe_7S_8/ FeS_2/NCNT

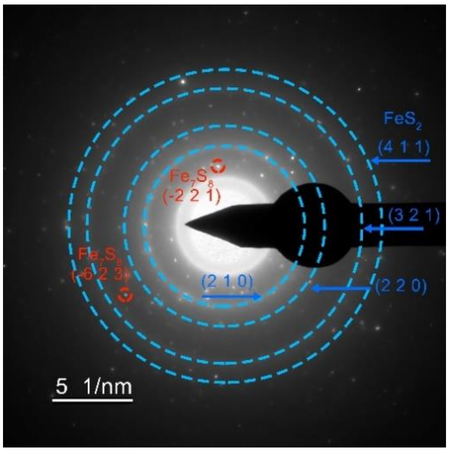

Fig. S3 SEAD pattern of Fe_7S_8/ FeS_2/NCNT

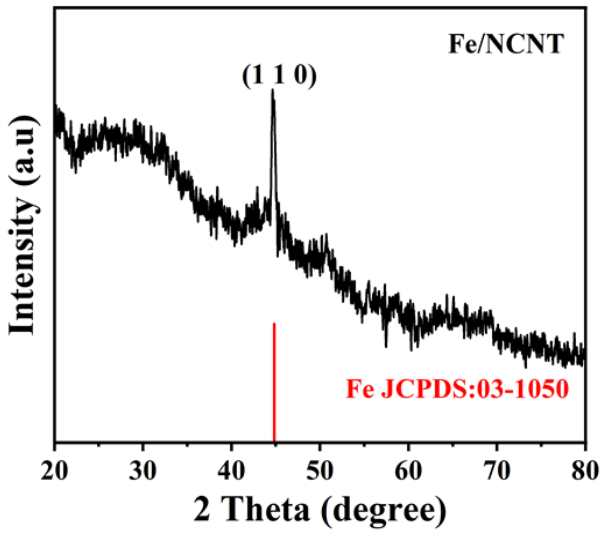

Fig. S4 XRD pattern of Fe/NCNT

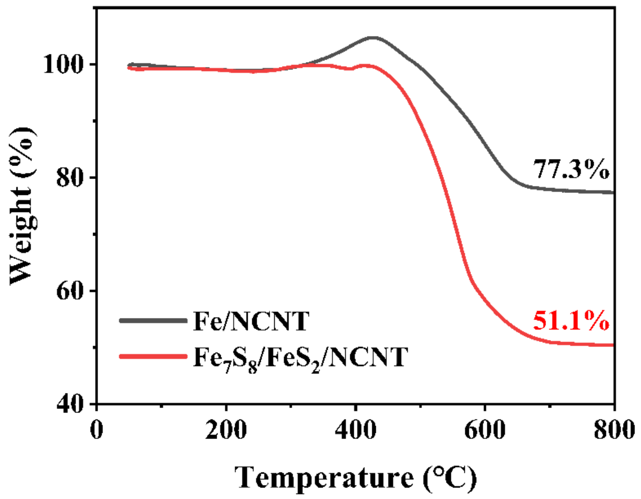

Fig. S5 TG curves of Fe_7S_8/FeS_2/NCNT and Fe/NCNT in air

For the Fe/NCNT, the content of Fe in Fe/NCNT is calculated to be 54.1 wt% based on the remaining 77.3 wt% of Fe_2O_3 after the TG test. Similarly, the the content of Fe in the Fe_7S_8/FeS_2/NCNT is calculated to be 35.8 wt%. Besides, since the sulfidation process will not change the mass ratio of Fe element to NCNT from Fe/NCNT to Fe_7S_8/FeS_2/NCNT, thus the content of NCNT in Fe_7S_8/FeS_2/NCNT is calculated to be 30.4% based on the following relationship:

$$W_{1\,Fe}/W_{1\,NCNT} = W_{2\,Fe}/W_{2\,NCNT}$$

where the $W_{1\,Fe}$ and $W_{1\,NCNT}$ correspond to the mass ratio of Fe element and NCNT in Fe/NCNT, $W_{2\,Fe}$ and $W_{2\,NCNT}$ correspond to the mass ratio of Fe element and NCNT in Fe_7S_8/FeS_2/NCNT.

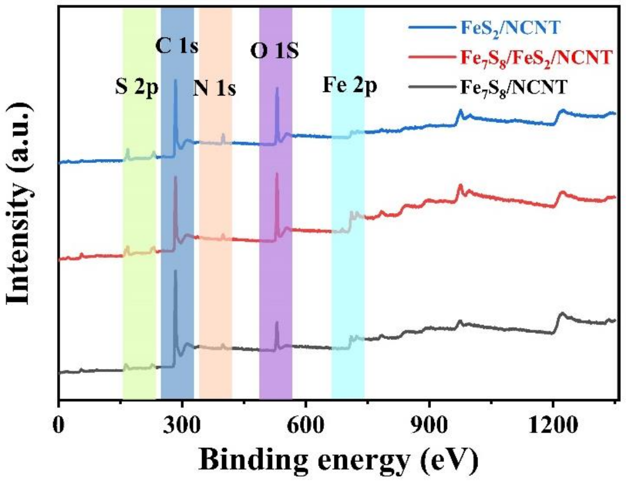

Fig. S6 XPS survey spectra of the Fe_7S_8/NCNT, Fe_7S_8/FeS_2/NCNT, and FeS_2/NCNT

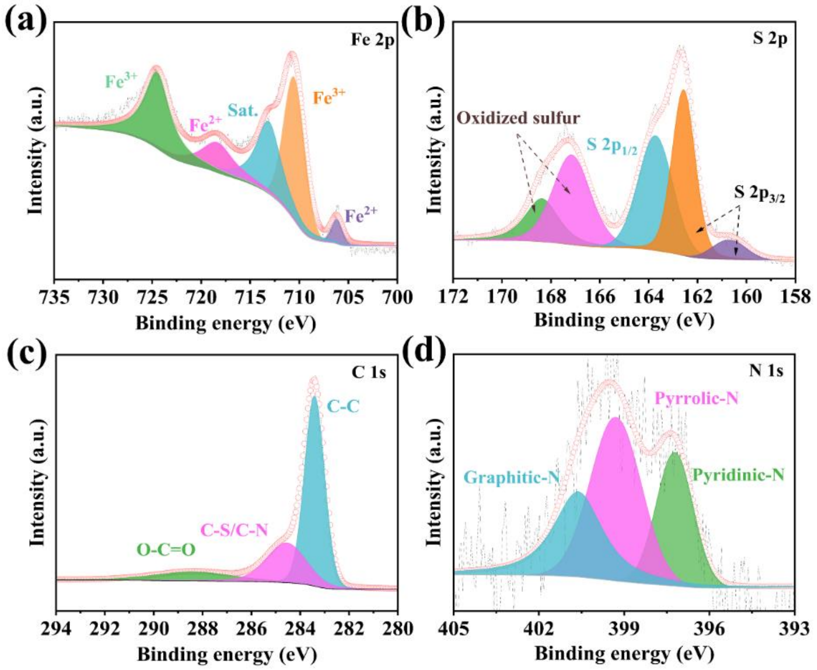

Fig. S7 High-resolution XPS spectra of Fe_7S_8/NCNT: **a** Fe 2p, **b** S 2p, **c** C 1s, and **d** N 1s

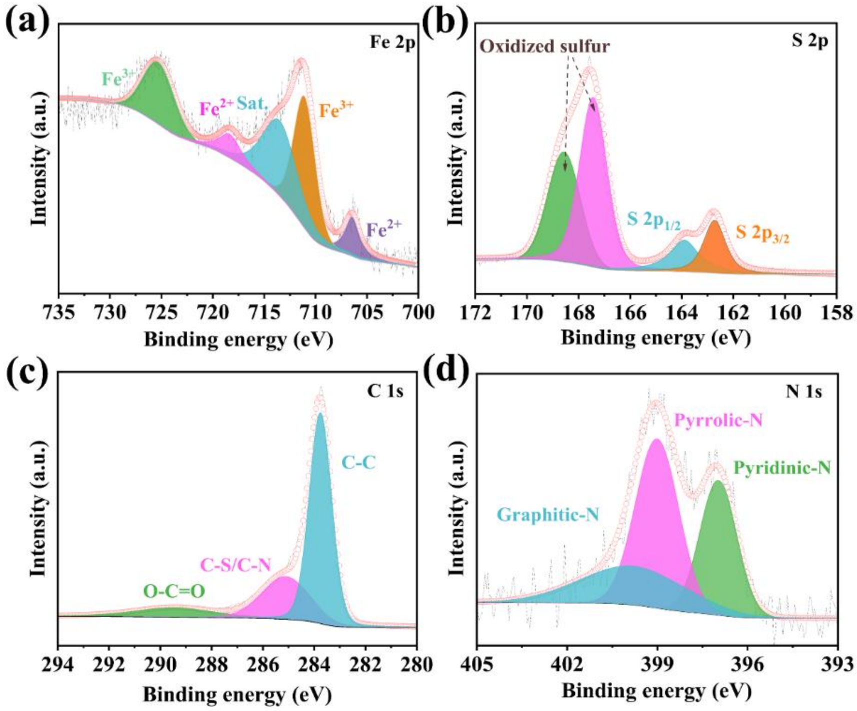

Fig. S8 High-resolution XPS spectra of FeS_2/NCNT: **a** Fe 2p, **b** S 2p, **c** C 1 s, and **d** N 1 s

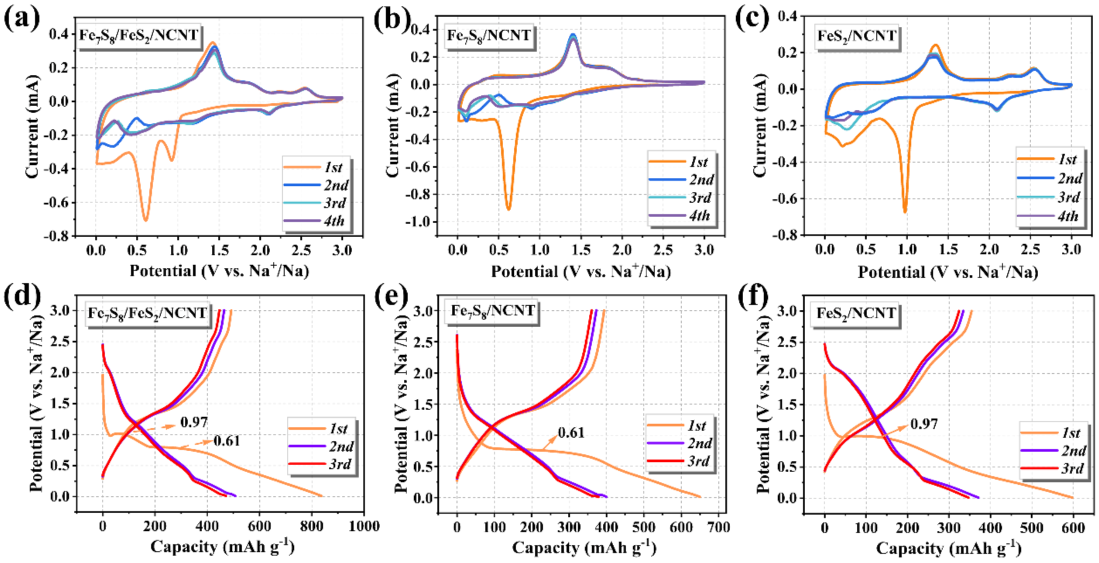

Fig. S9 CV curves of **a** Fe_7S_8/FeS_2/NCNT, **b** Fe_7S_8/NCNT, and **c** FeS_2/NCNT for the first four cycles at a scan rate of 0.2 mV s^{-1}. Charge/discharge voltage profiles of **d** Fe_7S_8/FeS_2/NCN, **e** Fe_7S_8/NCNT, and **f** FeS_2/NCNT for the first three cycles at a current density of 1.0 A g^{-1}

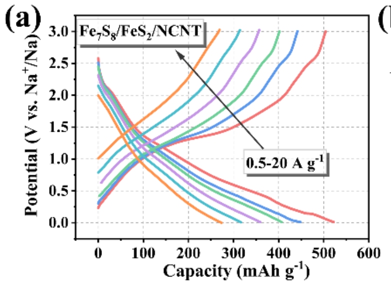

Fig. S10 Charge/discharge voltage profiles of **a** Fe_7S_8/FeS_2/NCNT, **b** Fe_7S_8/NCNT, and **c** FeS_2/NCNT at various current densities

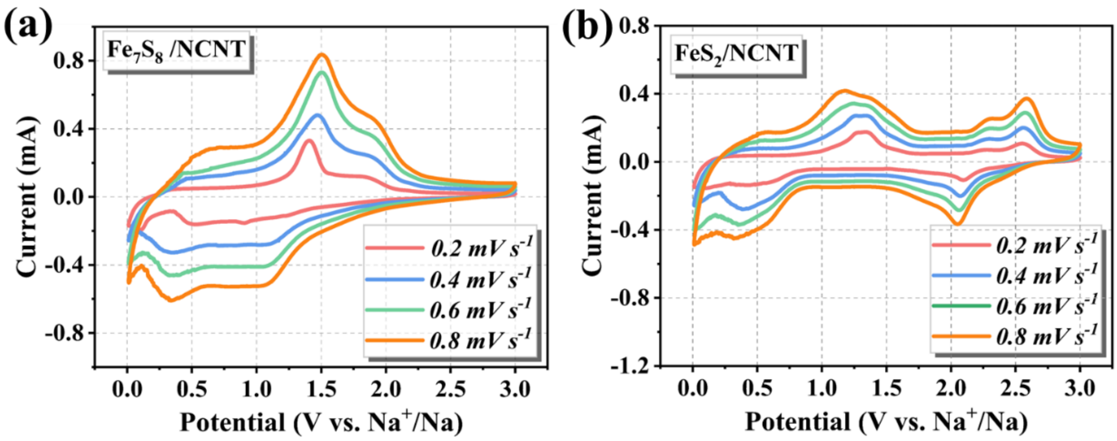

Fig. S11 CV curves of **a** Fe_7S_8/NCNT and **b** FeS_2/NCNT at scan rates ranging from 0.2 to 0.8 mV s^{-1}

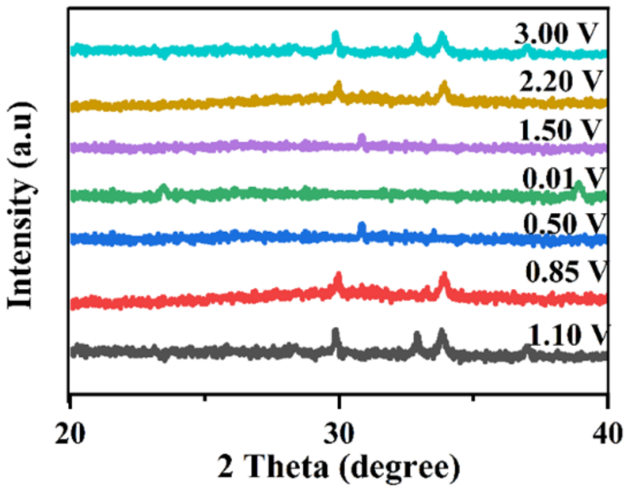

Fig. S12 Ex-situ XRD pattern of the Fe_7S_8/FeS_2/NCNT electrode at various charging/discharging states within the 2θ range from 20° to 40°

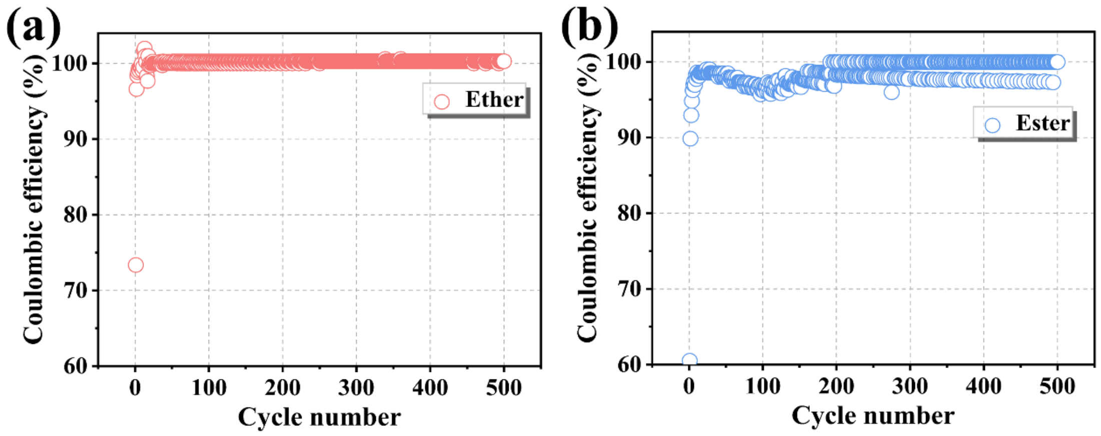

Fig. S13 Coulombic efficiency of electrodes circulating in **a** ether- and **b** ester- based electrolytes at 5 A g^{-1}

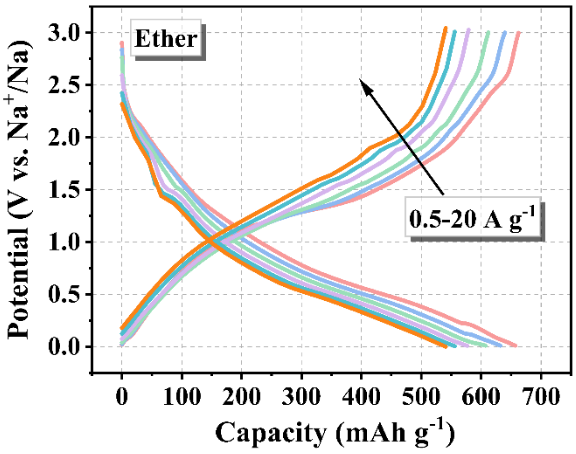

Fig. S14 Charge/discharge voltage profiles at various current densities of Fe_7S_8/FeS_2/NCNT in the ether-based electrolyte

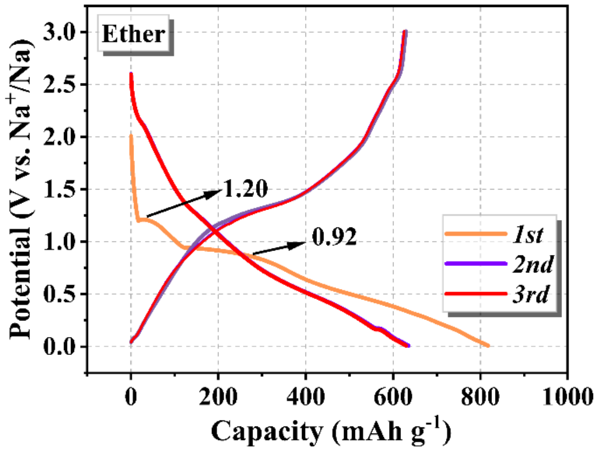

Fig. S15 Discharge/charge profiles at 1 A g^{-1} of Fe_7S_8/FeS_2/NCNT in the ether-based electrolyte

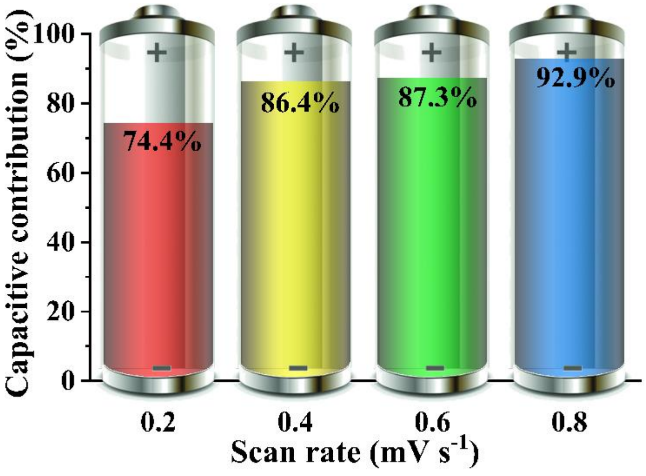

Fig. S16 Capacity contributions of the capacitive and diffusion-controlled behaviors at different scan rates in ester-based electrolyte

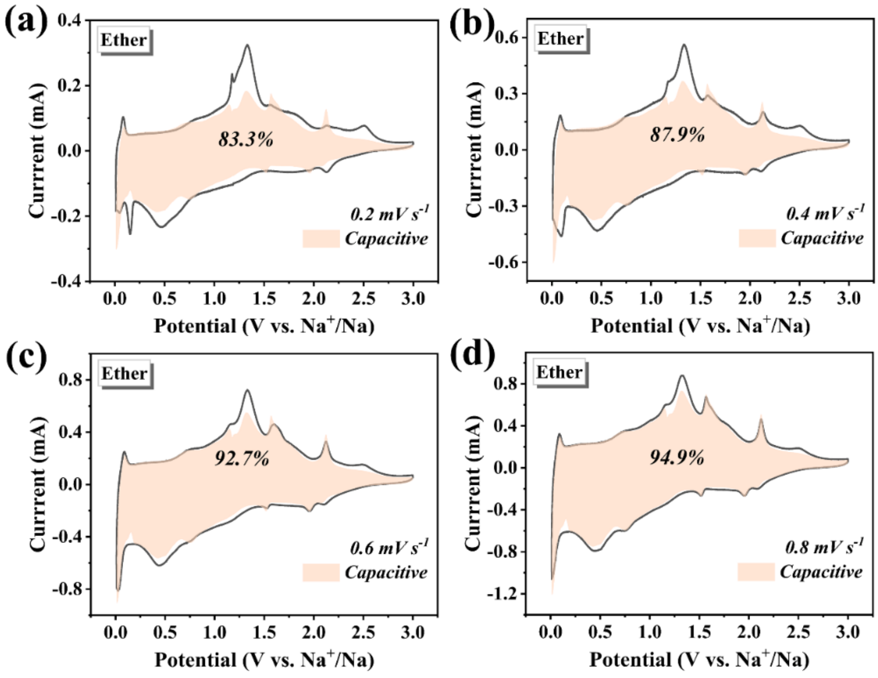

Fig. S17 Capacitive and diffusion-controlled contributions from 0.2 to 0.8 mV s^{-1} in ether-based electrolyte

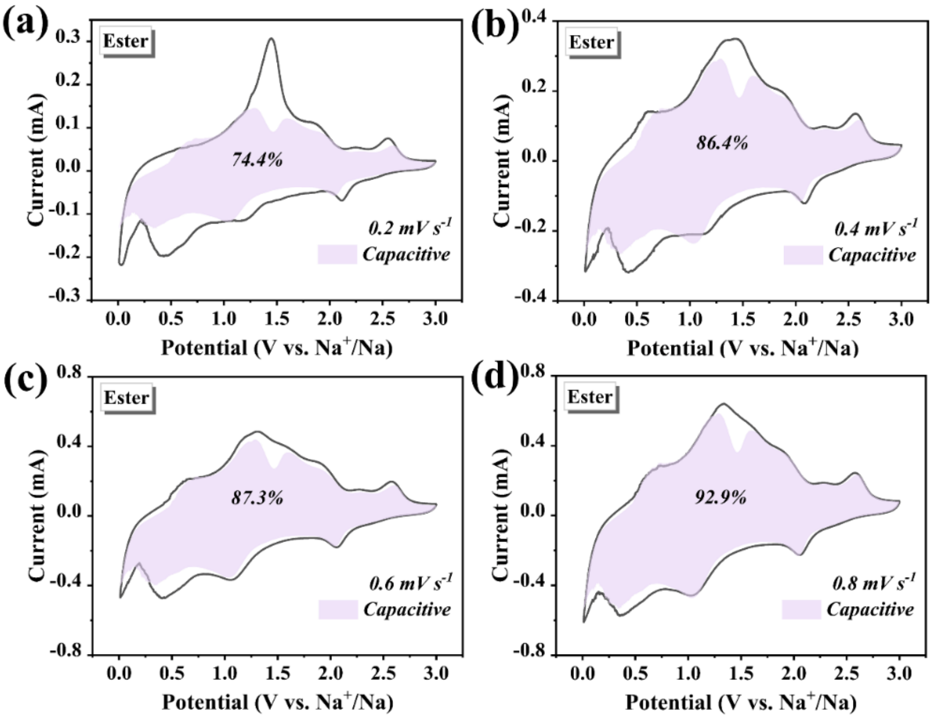

Fig. S18 Capacitive and diffusion-controlled contributions from 0.2 to 0.8 mV s^{-1} in ester-based electrolyte

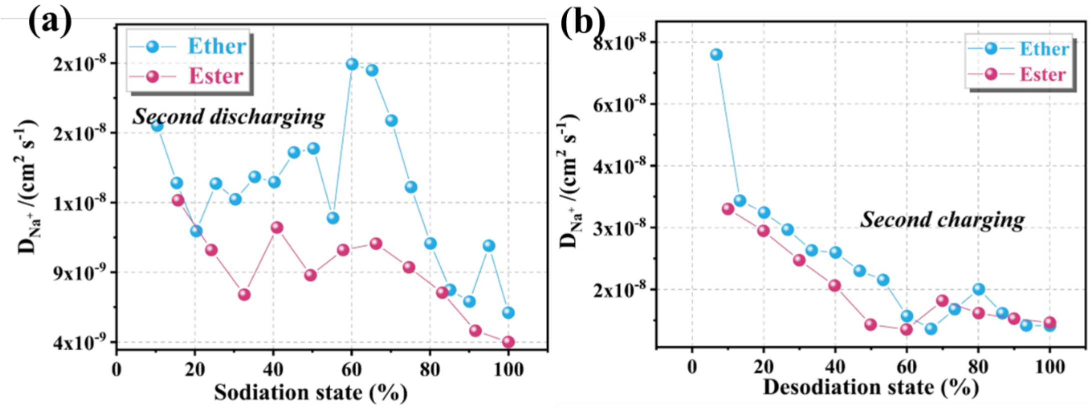

Fig. S19 Calculated Na^{+} diffusion coefficients upon the second **a** discharge and **b** charge in two different electrolytes

Table S1 Comparison of electrochemical performance of the as-fabricated Fe_7S_8/FeS_2/NCNT with other related electrode materials reported in the literature

Sample	Cut-off voltage (V)	electrolyte	Cycling stability: Current density (A g^{-1})	Cycle number	Capacity retention (mAh g^{-1})	Rate capability: Current density (A g^{-1})	Capacity retention (mAh g^{-1})	Refs.
**Fe_7S_8/FeS_2/NCNT**	**0.01-3.0**	**1 M $NaPF_6$ in diglyme**	**5.0**	**1000**	**466.7**	**10/20**	**556/537**	**This work**
Fe_7S_8@C NCs	0.08-3.0	1 M $NaPF_6$ in diethylene glycol dimethyl ether	0.18	1000	447	2.7	552	[S1]
Fe_7S_8@HD-C	0.01-3.0	1 m NaPF6 solution in DEGDME	2.0	320	480	5/10	401/326	[S2]
Fe_7S_8/N-C	0.01-3.0	1.0 M $NaClO_4$ in ethylene carbonate/propylene carbonate	0.2	500	451	1.6/3.2	353/328	[S3]
Fe_7S_8@C	0.01-3.0	1.25 M $NaPF_6$ in ethylmethyl carbonate	1.0	1000	531	2.0/5.0	558/537	[S4]
Fe_7S_8@C-G	0.01-3.0	1.0 M $NaClO_4$ in ethylene carbonate/propylene carbonate	0.1	100	478	1.0/2.0	332/306	[S5]
FeS_2@NC	0.5-3.0	1 M $NaClO_4$ in propylene carbonate	5.0	1000	375	5.0/10	407/307	[S6]
FeS_2/rGO	0.01-2.3	1 M $NaClO_4$ in ethylene carbonate/propylene carbonate	0.1	100	610	10	426/344	[S7]
FeS_2@C yolk–shell	0.1-2.0	1 M $NaSO_3CF_3$ in diethylene glycol dimethyl ether	2.0	800	330	2.0/5.0	470/403	[S8]
FeS_2/CNS	0.01-3.0	1 M $NaCF_3SO_3$ in diglyme	1.0	350	577	2.0/5.0	585/400	[S9]
FeS_2/ NHCFs	0.005-3.0	1 M $NaPF_6$ in diglyme	1.0	400	414	10/20	320/280	[S10]

Supplementary References

[S1] M. J. Choi, J. Kim, J. K. Yoo, S. Yim, J. Jeon et al., Extremely small pyrrhotite Fe_7S_8 nanocrystals with simultaneous carbon-encapsulation for high-performance Na-ion batteries. Small **14**(2), 1702816 (2018). https://doi.org/10.1002/smll.201702816

[S2] H. Li, Y. Ma, H. Zhang, T. Diemant, R. J. Behm et al., Metal-organic framework derived Fe_7S_8 nanoparticles embedded in heteroatom-doped carbon with lithium and sodium storage capability. Small Methods **4**(12), 2000637 (2020). https://doi.org/10.1002/smtd.202000637

[S3] A. Jin, M.-J. Kim, K.-S. Lee, S.-H. Yu, Y.-E. Sung. Spindle-like Fe_7S_8/N-doped carbon nanohybrids for high-performance sodium ion battery anodes. Nano Res. **12**(3), 695-700 (2019). https://doi.org/10.1007/s12274-019-2278-y

[S4] L. Shi, D. Li, J. Yu, H. Liu, Y. Zhao et al., Zhu. Uniform core–shell nanobiscuits of Fe_7S_8@C for lithium-ion and sodium-ion batteries with excellent performance. J. Mater. Chem. A **6**(17), 7967-7976 (2018). https://doi.org/10.1039/c8ta00985f

[S5] W. Huang, H. Sun, H. Shangguan, X. Cao, X. Xiao et al., Three-dimensional iron sulfide-carbon interlocked graphene composites for high-performance sodium-ion storage. Nanoscale **10**(16), 7851-7859 (2018). https://doi.org/10.1039/c8nr 000 34d

[S6] R. Zang, P. Li, X. Guo, Z. Man, S. Zhang et al., Yolk-shell N-doped carbon coated FeS_2 nanocages as a high-performance anode for sodium-ion batteries. J. Mater. Chem. A **7**(23), 14051-14059 (2019). https://doi.org/10.1039/C9TA03917A

[S7] Q. Wang, C. Guo, Y. Zhu, J. He, H. Wang. Reduced graphene oxide-wrapped FeS_2 composite as anode for high-performance sodium-ion batteries. Nano-Micro Lett. **10**(2), 30 (2018). https://doi.org/10.1007/s40820-017-0183-z

[S8] Z. Liu, T. Lu, T. Song, X.-Y. Yu, X. W. Lou et al., Structure-designed synthesis of FeS_2@C yolk-shell nanoboxes as a high-performance anode for sodium-ion batteries. Energy Enviro. Sci. **10**(7), 1576-1580 (2017). https://doi.org/10.1039/c7ee01100h

[S9] Z. Lin, X. Xiong, M. Fan, D. Xie, G. Wang et al., Scalable synthesis of FeS_2 nanoparticles encapsulated into N-doped carbon nanosheets as a high-performance sodium-ion battery anode. Nanoscale **11**(9), 3773-3779 (2019). https://doi.org/10.1039/c8nr10444a

[S10] C. Zhang, D. Wei, F. Wang, G. Zhang, J. Duan et al., Highly active Fe_7S_8 encapsulated in N-doped hollow carbon nanofibers for high-rate sodium-ion batteries. J. Energy Chem. **53** 26-35 (2021). https://doi.org/10.1016/j.jechem.2020.05.011
